# Effectiveness of Integrated Social and Behavior Change Communication Interventions in Mass Drug Administration Campaigns in Enhancing Knowledge, Perceptions, and Preventive Practices for Neglected Tropical Diseases in Jimma

**DOI:** 10.2147/RMHP.S468390

**Published:** 2024-10-01

**Authors:** Daba Abdissa, Yohannes Kebede, Sudhakar Morankar, Gelila Abraham, Gebeyehu Bulcha, Teshome Shiferaw, Nimona Berhanu, Firanbon Teshome, Hirpa Miecha, Zewdie Birhanu

**Affiliations:** 1 Department of Biomedical Sciences, Jimma University, Jimma, Ethiopia; 2 Department of Health, Behavior and Society, Jimma University, Jimma, Ethiopia; 3 Department of Health Policy and Management, Jimma University, Jimma, Ethiopia; 4 Jimma Zone Health Office, Oromia, Ethiopia; 5 School of Pharmacy, Jimma University, Jimma, Ethiopia; 6 Oromia, Regional Health Bureau, Oromia, Ethiopia

**Keywords:** Knowledge, Perception, Preventive practices, Effectiveness, SBCC, Jimma, Ethiopia

## Abstract

**Background:**

Despite control efforts, including mass drug administration (MDA), neglected tropical diseases (NTDs) continue to pose a significant public health threat, particularly in rural Ethiopia. Integrating social and behavior change communication (SBCC) into MDA is essential for success. This study aimed to evaluate the effectiveness of tailored SBCC integrated into MDA campaigns to enhance community knowledge, perception, and preventive behaviors regarding targeted NTDs in the Jimma, Ethiopia.

**Methods:**

A community-based study was conducted using a pre-test post-test design. A multistage sampling technique was employed for surveys, while for qualitative insights, purposive sampling was employed. SBCC interventions tailored to local needs were implemented. Changes in knowledge, perception, and practices were evaluated using Cohen’s d. Additionally, a score for key outcome variables comparisons were made to examine variations based on socio-demographic factors, employing suitable statistical tests. Qualitative data were analyzed thematically using Atlas.ti 7.1.5.

**Results:**

The prevalence of SBCC exposure was 88.8%. The intervention had a more significant impact on improving knowledge and preventive practices related to Onchocerciasis (OC) compared to Soil-transmitted helminthes (STH). Specifically, most OC outcomes showed moderate to large effect sizes. In contrast, the effect on STH was more limited, with only knowledge of consequences improving moderately and preventive practices showing a small effect size. The mean score for OC preventive practices varied by educational level and sex, while the median score for STH perception varied among educational level and marital status. Additionally, score variation was observed across districts for all key outcome variables.

**Conclusion:**

The majority of the population was exposed to SBCC resulting in improvements in knowledge, perception, and preventive behaviors regarding OC and knowledge of consequences of STH and its prevention behavior. This highlights the importance of incorporating well-designed SBCC activities in to MDA campaigns to optimize the control and eventual elimination of targeted NTDs.

## Introduction

Neglected Tropical Diseases (NTDs) are a group of diseases that predominantly affect rural populations in tropical regions, posing a significant public health challenge. Despite control efforts, these diseases continue to persist. The Ethiopian Federal Ministry of Health has identified nine priority NTDs, including Onchocerciasis(OC) and Soil-Transmitted Helminthes (STH).^1^ Control efforts for NTDs include Mass Drug Administration (MDA), vector control, improving water, sanitation and hygiene (WaSH) and health education to promote safe practices and early diagnosis. These diseases are associated with poverty and are prevalent in regions with inadequate safe water supplies, inadequate sanitation, and poor housing. An estimated 1 billion people worldwide are thought to be affected by at least one NTD, which can lead to major health issues as well as social and economic hardship.^2^

Soil transmitted helminthes are the most predominant NTDs. As per the Global Burden of Diseases (GBD) 2016 report, they affect 1.5 billion people globally, with the highest burden found in sub-Saharan Africa.^3–5^ Another NTD that commonly results in severe illness in low-income countries is OC, and approximately 99% of OC diseases take place in rural areas of sub-Saharan Africa, close to rivers.^6^ Furthermore, according to the GBD, there were 20.9 million OC infections in 2017.^7^

In Ethiopia, STH represent a significant public health challenge, affecting over 79 million people.^8–10^ It is endemic to 89% of the country’s districts^11^ and its prevalence ranges from 18.1% to 70.3%.^12–14^ The primary ways in which these parasites spread are through the larvae and the release of eggs into human feces, which contaminate the environment in places with inadequate sanitation and hygiene.^15^ STH infections have far-reaching consequences, impacting individuals and communities in multiple ways. They hinder children’s learning and adults’ productivity by depleting energy and causing delayed physical growth, school absenteeism, and bowel obstruction.^16^ Additionally, STH infections lead to malnutrition, impaired cognition, and adverse pregnancy outcomes. The cumulative effect of these consequences is to reduce economic output, ultimately trapping endemic communities in a cycle of poverty.^4^,^17^

In Ethiopia, 188 districts are endemic to OC^18^ and it is a pressing public health problem in spite of many control measures. It is transmitted through the repeated bites of infected Simulium blackflies. It is particularly prevalent in the large-scale coffee plantation areas of southwest Ethiopia, which are highly populated, heavily forested, and contain many perennial rivers and streams.^19^ The adult female worm discharges numerous mobile microfilariae daily, which travel beneath the dermis of the skin, leading to significant inflammation in the affected region.^20^ The disease causes significant morbidity, psychological problems, diminished productivity, and blindness.^21^,^22^

Following WHO guidelines, Ethiopia initiated MDA campaign with complementary interventions to eliminate both diseases as a public health concern by 2030.^23^ Nevertheless, in many endemic settings in Ethiopia, treatment coverage is below optimal^24–26^ because of several factors. These include misperceptions about the disease,^27^,^28^ poorly coordinated interventions, failure to adequately integrate MDA-related interventions with other interventions.^29–31^ Furthermore, the STH MDA targets particular risk groups, has a transient effect on transmission, cannot prevent reinfection, and does not destroy immature worms. Consequently, after successful MDA treatment, human reinfection occurs rapidly.^24^ This indicates that in order to achieve the goal it will be necessary to develop an effective plan and strategies for the long-term control and eradication of both diseases by implementing the necessary complementary interventions. Complementary interventions for OC control include vector control through personal protection measures against biting insects, treatment of cases, and awareness-raising activities to prevent bites.^25^ For STH control, provision of safe WASH infrastructure and practices is crucial, along with health education programs to promote healthy behaviors and prevent infection.

In the context of eliminating NTDs, one of the key interventions is to improve and maintain sufficient levels of knowledge and appropriate preventive practices among the public.^26^ Understanding the knowledge, perception, and practices of a community is important to improve the prevention and control of NTDs. This can be achieved by strengthening control measures that are locally accepted through strategies tailored to fit cultural beliefs and practices, ensuring community engagement and ownership.^32^,^33^ This is because the accurate understanding and perceptions of the community, as well as their adoption of preventive actions, play a vital role in the efficacy and effectiveness of NTD control measures. Individuals who perceive higher risks associated with the diseases are more inclined to take protective actions, which contributes to the overall success of NTD control efforts.^32–34^

One of the recommendations from a recent scoping review on NTD interventions emphasizes the importance of addressing social and ecological determinants of NTDs.^35^ Many of these diseases are preventable, and could be eliminated with various public health, health promotion and medical interventions. Implementing SBCC is a key strategy for improving community understanding of targeted NTDs. This approach involves the systematic use of interactive, theoretically grounded, and evidence-based methods by using of any communication opportunity and to encourage changes in knowledge, attitudes, norms, beliefs, and behaviors.^36^ When combined with other interventions, SBCC is cost-effective and efficient,particularly for underprivileged rural communities to encourage appropriate knowledge and preventive behaviors linked to targeted NTDs.^37^,^38^ Furthermore, evidence has demonstrated that SBCC is key to achieving many public health goals for preventing and managing a variety of public health issues and approaches the problem from several perspectives including community engagement, behavior change, and education.^39^,^40^ Health education and communication are critical to all facets of health promotion and disease prevention because they raise awareness, alter perceptions, and reinforce behavioral changes.^41^,^42^

The Ethiopia National Health Promotion and Communication Strategy Framework (2016–2020) indeed recognized the crucial role of awareness, behavior change, and social mobilization in achieving health improvements in the country. By emphasizing the importance of information, communication, social mobilization, and advocacy, the framework acknowledged that health outcomes are closely tied to the knowledge, attitudes, and practices of individuals and communities.^43^ However, health communications in Ethiopia have generally not been prioritized compared to other health-related activities.^44^

In Ethiopia, a number of MDA campaigns are conducted annually, with the integration of SBCC interventions being essential to their success. There is significant demand and expectation for effective incorporation of tailored SBCC into these MDAs campaigns. However, there are gaps in incorporating educational components into these campaigns, and there is a lack of evidence on the effectiveness and optimal methods of integrating SBCC into these campaigns.^45^ Therefore, the purpose of this study was to evaluate the effectiveness of tailored SBCC integrated into MDA campaign of targeted NTDs on knowledge, perceptions, and preventive practices concerning targeted NTDs in the Jimma Zone of Oromia, Ethiopia. The results of this study contribute to the success of control measures for targeted NTDs in the study area and similar settings.

## Methods and Materials

### Study Area, Population, Period and Design

A community based study employing a mixed-methods approach was done using a pre-test–post-test design in Jimma zone, Ethiopia. The zone is situated approximately 357 kilometers to the west of Addis Ababa, countries’ capital city. A total of five districts were chosen from the 22 districts in the zone, based on local NTD expert input and careful consideration of factors such as disease endemicity (Supplementary Figure 1). Both diseases are endemic in the selected districts. According to government census data, the prevalence of STH in the Jimma Zone is estimated to be between 20–30%. This is further supported by a recent study among adult residents in the peri-urban areas of Jimma, which reported STH prevalence of 18.1%.^14^,^46^ Regarding OC, an entomological survey conducted by the Ethiopian Public Health Institute found a high prevalence of blackfly vectors and microfilarial load in the Jimma Zone, indicating a substantial burden of OC in the region.^46^

The baseline data were collected in October and November 2021, whereas endline data collected in June and September 2022. Our study populations were primarily spouses of heads of households who had resided in the study area for at least 6 months. For qualitative part diverse range of participants were included, including volunteers, community members, primary healthcare unit (PHCU) leaders, NTD and WaSH experts, and health extension workers to ensure a comprehensive understanding of the issue.

### Sample and Procedure

This study was part of a larger study aimed to “evaluate the effectiveness, feasibility and acceptability of co-delivery of two MDA for OC and STH”. The sample size was determined using single proportion formula using 75% (effective campaign treatment coverage of STH) and considering design effect of 1.5, margin of error 4% and 10% non-response rate which gave 743. To ensure representative sampling multistage sampling was employed, initially, five districts within the Jimma Zone were selected based on input from local health experts and consideration of the targeted endemicity of NTDs. Then two gandas, which are the lowest administrative units in Oromia, Ethiopia, were randomly selected from each of the selected districts. Lastly, a simple random sampling technique was utilized to select participants at the household level. The details (study area, population, sample size determination and procedure) have been described in our previous study.^47^

Purposeful sampling technique was employed for the qualitative portion of the study and to ensure data saturation. Data collection continued until daily reviews and preliminary analyses indicated that little new information was emerging. The recruitment process for participants took into account various factors, including the setting, gender, experience, and position. Accordingly, nine key informant interviews (KIIs), four focus group discussions (FGDs) (each consisting of six to 12 participants), and four expert group discussions (EGDs) (each consisting of two or three participants) were conducted. The FGD participants were youth, volunteers, and female adult community members, whereas the KII participants were health extension workers, volunteers, PHCU leaders, and NTD and WaSH experts, focal at the district level. The EGD participants were NTD and WaSH experts at the district level and HEWs.

### Intervention Packages and Procedure

Based on the insights gathered from the formative assessment, which combined both quantitative and qualitative data, as well as existing resources, a set of well-designed and harmonized health education messages were developed. These messages were carefully crafted to be locally sensitive and acceptable, taking into account the unique cultural, social, and environmental context of the target communities. These messages focused on creating understanding, motivation, and attitudinal change for adopting healthy behaviors and leveraging the integrated campaign platforms of STH and OC. It was implemented before and during the campaign for targeted NTDs.

The education was aided by locally appropriate SBCC materials (posters, integrated brochures, information, education and communication cards, banners, and flipcharts) and a harmonized training manual. Before production, the materials were sufficiently pretested to ensure their understandability and relevance, and to convey appropriate information. In addition, local media, such as the public crier, were used to transmit key messages to the community. Key SBCC educational contacts and approaches implemented to increase the reach and frequency of exposure to messages are described in the following.

#### First SBCC Exposure

During house-to-house visits for community registrations to determine the eligible target for the proposed MDA, households received key messages on sanitation, hygiene, OC and STH by trained community volunteers. These volunteers were educating their families at the end of their registrations using a flipchart. They also distributed IEC information cards to households containing key information such as consequences, mode of transmission and preventive measures of targeted NTDs supported with key supporting explanatory figures.

#### Second SBCC Exposure

As part of community mobilization, during the pre-campaign and intra-campaign the community volunteers, frontline health workers, and community leaders including religious leaders delivered key messages to community members, at community gatherings and mosques/churches. The main task of this team were to inform, sensitize, and disseminate key SBCC messages, and educate and mobilize (re-mobilize) communities to ensure effective community engagement for campaigns and increase their understanding of targeted NTDs. For consistency and focus, the volunteers were guided by a single written key message outlined in the local language for announcements. Brochures were distributed to the general community who could read, and posters were fixed at public gathering places where people could read and understand. Banners, posters, and message cards were used for the announcements. Community volunteers were remobilized to ensure attendance based on the daily performance of MDA. Health workers from the PHCU supported HEWs in addition to supervisory and overall coordination.

#### Third SBCC Exposure

Key SBCC messages were disseminated through schools, using banners and posters. School communities participated in key information dissemination and facilitated the participation of students in receiving MDA drugs in nearby communities together with their parents. School-based key education messages were given by experts from the research team and trained community volunteers, and the students acted as messengers to reach out to their families and other community members.

#### Final SBCC Exposure

During the campaign, information dissemination and counseling of participants was conducted by HEWs, volunteers, PHCU health workers, and others. Upon the arrival in communities during the campaign, they received health education and key messages on targeted NTDs, services they were going to receive, benefits/purposes, ways to prevent these diseases using posters and flipped charts, and displayed banners. During the campaign event, a larger poster promoted appropriate hygiene behaviors (sanitation practices such as toilet usage, hand hygiene, water handling, treatment, etc). A separate larger poster addressing key facts and illustrative behavioral actions was displayed at the campaign delivery site and explained by a trained frontline health worker in small groups. Appropriate hand washing practices were demonstrated at the campaign site. To reinforce the message received at the campaign delivery site, tailored brochures were shared with parents (one per household) and encouraged to be read at home or for someone in the family to read it loudly to household members. The brochures were designed such that they contained key messages related to the targeted NTDs, MDA and sanitation/hygiene.

### Theoretical Basis of the Study

The study was guided by the RE-AIM framework, which has five main constructs.^48^,^49^ In this framework, one of the effectiveness components was changes in the community’s perception, knowledge, and healthy practices regarding targeted NTDs as a result of tailored SBCC, which was integrated into the MDA program. Furthermore, one of the reach components in the framework is the proportion of the target population that has been exposed to social and behavior change communication (SBCC) information in terms of source and message content. The implementation component refers to the extent to which the active ingredients of the interventions were successfully delivered with fidelity according to the established research protocol. For each program activity, the percentage of output (planned vs output) was calculated to yield the level of adherence to the proposed specifications, both in terms of the package of services and the timelines and adaptation strategies implemented.

### Data Collection Tool and Procedure

Data were collected using a structured interviewer-guided questionnaire, which was developed based on relevant literature and findings from qualitative studies. Endline data were collected following the intervention using the same tool as at baseline. In the qualitative part of the study, a semi-structured guide was utilized. This guide was developed by reviewing relevant literature and taking into account the research objectives. Skilled experts at the master’s level, who were fluent in the local language, conducted the data collection. The interviews or discussions were recorded using a digital voice recorder to ensure accurate capture of the information shared by the participants.

### Measurements and Operational Definitions

#### Knowledge on OC

To assess the multidimensional knowledge of OC, yes/no items were used. Each item in the questionnaire was assigned a score of 1 for a correct response and a score of 0 for an incorrect response. Separate indices were generated for each knowledge dimension by summing the scores of relevant items. The overall knowledge score was computed by summing the scores from all aspects of knowledge. Likewise, the measurement of risk perception utilized a five-item scale with a three-point response format: agree, disagree, and neither/do not know. Each participant’s response of “agree” was assigned a score of 1, while all other responses received a score of 0. These scores were then summed across the five items to generate a risk perception score for each participant.

To standardize and facilitate comparison across different scales, all variables (overall knowledge, its dimensions and risk perception) were rescaled to a range of 0 to 10 using the formula Y = (X–Xmin) * n / Xrange. In this formula, Y represents the rescaled variable, X denotes the original variable, Xmin represents the minimum observed value of the original variable, Xrange represents the difference between the maximum and minimum scores on the original variable, and n represents the upper limit of the rescaled variable (which is 10 in this case). By applying this rescaling formula, the original variables were transformed to a common scale of 0 to 10, allowing for easier comparison and interpretation. After rescaling, the levels of knowledge and perception were categorized as high if they exceeded 50% of the total adjusted score. Conversely, scores below this threshold were categorized as low, indicating a relatively lower level of knowledge or perception.

To measure OC preventive practices, a set of five yes/no relevant items were used. For each item, respondents were asked whether they engaged in the preventive practice or not. If the respondent reported using at least one of the preventive measures, their response was considered as “yes” indicating adherence to preventive practices. On the other hand, if the respondent did not report using any of the preventive measures, their response was considered as “no” indicating a lack of preventive practices. The details of measurement were reported in our earlier study.^47^

Regarding STH we followed similar process like in OC above. To assess the multidimensional knowledge, a set of pertinent yes/no items was used. Accordingly, there were seven items on symptoms, eight items on the mode of transmission, five items on the consequences, and ten items on preventative measures. Similarly, STH preventive practices were measured using nine yes/no items and the scores were summed to give preventive practice score. Each item in the questionnaire was assigned a score of 1 for a correct response and a score of 0 for an incorrect response. Separate indices were generated for each knowledge dimension by summing the scores of relevant items. The overall knowledge score was computed by summing the scores from all aspects of knowledge like that of OC. Finally, the perceived risk toward STH was measured using three items with a three-point response format (agree, disagree, do not know). To compute the score, each response with agreement was recoded as yes and scored 1 point; and otherwise 0 points. The scores were then summed to obtain risk perception scores.

To enable standardization and comparison across different scales of STH measurement, all the measurement scales used in the study, including those for knowledge, perception, and practice were rescaled to a range of values from 0 to 10, similar to the rescaling method used for OC. After rescaling, the levels of knowledge, perception, and practice were categorized based on their scores. If a participant’s score was above the mean score, it was considered as a high level in that particular category (knowledge, perception, practice). Conversely, scores below the mean indicated a low level in that category.

#### Frequency of Hand Washing Practice at Critical Times

Frequency of hand washing practice at critical times were measured using eight Likert-scale questions (never, sometimes, often, and always), with a minimum score of 8 and a maximum score of 32. An adequate frequency of hand washing practice was considered if the score was exceeded three-quarters of the total score; otherwise, inadequate practice.

#### Household Water Treatment Practice

Household water treatment practice was assessed using five yes/no items and coded as 1 if the household practiced at least one of the water treatment methods (adding medicine, boiling and cooling, filtering using clothes, using a water filter, and adding lemons), and as 0 if not.

#### Adequate Knowledge on Perceived Benefit Toilet Use

This study used five yes/no items (protecting the family’s health, preventing communicable diseases, preventing flies, preventing water contamination, and maintaining environmental and personal hygiene) and considered them adequate if they exceeded 50% of the total score.

#### Proper Hand Washing Knowledge

Act of washing hands with water and soap or ash at critical times.

#### Exposure to SBCC Messages

Respondents were asked whether they received any information or message on targeted NTDs during all four opportunities for SBCC exposure described under the intervention package, with follow-up questions to capture the source and content of the message they received.

### Data Processing and Analysis

Following data collection, the data was checked to ensure completeness and accuracy. The validated data was then entered into Epidata version 4.6, and subsequently exported to SPSS version 26.0 for further statistical analysis. At each step, appropriate coding and re-coding of variables were performed as required to facilitate accurate and meaningful analysis. After standardizing the scores of the variables, descriptive statistics were computed to summarize and describe the findings of the study.

To examine the changes in standardized scores of knowledge, perception, and practices between the baseline and endline surveys, Cohen’s d was utilized to calculate the magnitude of the effect size. We used the following benchmarks for effect size interpretation: d < 0.2 very small effect, 0.2 ≤ d< 0.5 represents a small effect, 0.5 ≤ d< 0.8 denotes a medium effect and d ≥ 0.8 suggests a large effect.^50^ Furthermore, score differences for key outcome variables, including knowledge, perception, and practice scores, were calculated by subtracting endline scores from baseline scores. To examine variations in these differences based on socio-demographic factors, independent sample *t*-tests and one-way ANOVA were utilized for normally distributed data. For non-normally distributed data, median comparisons were conducted using the Mann–Whitney *U*-test and Kruskal–Wallis test.

The qualitative part of the study involved transcribing the audio data verbatim and translating it into English. The investigators employed Atlas.ti 7.1.5 software to code and conducting subsequent analyses. The transcripts were thoroughly read and reviewed by the investigators, who assigned codes to all the transcripts. An inductive thematic analysis approach was adopted, which involved identifying patterns and themes that emerged from the data itself, rather than imposing preconceived categories. The data were coded, categorized, and organized into meaningful themes that captured the essence of participants’ responses. Relevant and representative direct quotes from the transcripts were selected to explain, confirm, and clarify the quantitative results, providing a richer understanding of the findings.

### Data Quality Assurance

Prior to commencing data collection, the data collectors received training on various aspects, including the techniques of data collection, the purpose of data collection, and the content of the questionnaire. They were also provided guidance on how to approach the respondents during the data collection process. Throughout the data collection phase, close supervision was maintained to ensure the quality and consistency of data collection activities. The tool was reviewed by experts to ensure its face and content validity. Before the actual data collection, a pretest was carried out to assess the suitability, clarity, and flow of the instrument in the local context. Any required modifications were implemented, and strict monitoring was employed to ensure adherence to the intervention protocol during the intervention.

Various techniques were employed to ensure the qualitative findings were dependable, credible, transferable, and confirmable. Throughout each interview and discussion, the facilitators provided summaries of the main points discussed. At the end of each session, participants were invited to give feedback or share additional insights. This process allowed for participant validation and ensured their perspectives were accurately captured. The transcripts were shared with colleagues for their input, and their feedback was carefully considered. An audit trial was conducted to validate the study findings, ensuring that the results were logical and derived from the data and to enhance transferability, the entire research process, was described in detail.

## Results

### Socio-Demographic Characteristics

The survey included a total of 1508 households, with 732 and 776 households participating at the baseline and endline, respectively. The mean age of the respondents at the baseline was 37.6±13.2 years, while at the endline, it was 37.3±12.9 years. The majority of participants at the endline were female (76.1%) and worked in farming (94.9%). Additionally, most (61.8%) had no formal education (Table 1).

**Table 1:** Socio-Demographic Characteristics of Respondents, Jimma Zone, Ethiopia, 2021–22

Characteristics	Category	Repeated Surveys				Combined	
		Baseline		Endline			
		No	%	No	%	No	%
Study district	Omo Nada	187	25.5	173	22.3	360	23.8
	Omo Beyam	131	17.9	149	19.2	280	18.6
	Kersa	137	18.7	147	18.9	284	18.8
	Gomma	162	22.1	157	20.2	319	21.2
	Manna	115	15.7	150	19.3	265	17.6
Sex	Male	137	18.7	224	28.9	361	23.9
	Female	595	81.3	552	71.1	1147	76.1
Marital status	Married	646	88.3	651	83.9	1297	86
	Widowed	43	5.9	61	7.9	104	6.8
	Others^a^	43	5.9	64	8.2	107	7.1
Role in household	Housewife	576	78.7	541	69.7	1117	74.1
	Husband	124	16.9	182	23.5	306	20.3
	Member	32	4.4	53	6.8	85	5.6
Education status	No formal education	409	55.8	523	67.4	932	61.8
	Primary	277	37.8	195	25.1	472	31.3
	Secondary	46	6.3	58	7.5	104	6.8
Religion	Muslim	638	87.2	703	90.6	1341	88.9
	Orthodox	66	9.0	59	7.6	125	8.3
	Others^b^	28	3.8	14	1.8	52	3.4
Ethnicity	Oromo	648	88.5	714	92.0	1362	90.3
	Amhara	31	4.2	29	3.7	60	3.9
	Others^c^	53	7.2	33	4.3	86	5.7
Occupation	Farmer	689	94.1	742	95.6	1431	94.9
	Other^d^	43	5.8	34	4.4	77	5.1

**Notes**: ^a^Baseline: 28 single, 6 separated, 9 divorced; endline: 41 single, 16 separated, 6 divorced, 1 other.^b^Baseline: 26 protestant, 2 other; endline: 14 protestant. ^c^Baseline: 25 hadiya, 9 Dawuro, 4 Yem, 8 Kafa, 7 other; endline: 14 Dawuro, 10 hadiyya, 6 Kaffa, 3 other.^d^Baseline: 8 private business, 13 daily laborer, 1 NGO worker, 3 pastoralist; endline: 19 daily laborer, 13 private business, 1 NGO worker, 1 governmental employee; No=number.

### SBCC Exposure During the Intervention

Overall, 88.8% (95% CI: 86.7, 90.9) of the survey respondents reported exposure to SBCC information. Most of them received education on OC drug benefits and how to take (67.4%) and benefits of taking OC and STH drugs together (44.6%), with the major sources of information being HEWs (66.7%) and volunteer youth (64.7%) (Table 2).

**Table 2:** SBCC Exposure and Its Source During the Intervention in the Jimma Zone, 2021–22

Information Heard			Source of Information		
About	(n=yes)	%	From	(n=yes)	%
OC drug benefits and how to take	522	67.4	Health extension workers	478	66.1
Benefits of taking OC and STH drugs together	345	44.6	Volunteers	468	64.7
Benefits and how to take germ drugs	322	41.6	Community meeting	53	7.3
OC disease: transmission, prevention and severity	139	18.0	Printing materials at home	45	6.2
Side effects of taking OC and STH drugs together	115	14.9	Students	37	5.1
STH: transmission, prevention and severity	113	14.6	Where drug distribution campaigns were conducted	35	4.8
Benefits and how to keep the environment clean	109	14.1	Head of gare/zoni/kebelle	34	4.7
Benefits and how to maintain personal hygiene	96	12.4	Health workers	26	3.6
COVID-19: transmission, prevention and severity	71	9.2	Printed materials on site/outside home	20	2.8
Benefits and methods of childhood vaccination	41	5.3	Religious leaders	4	0.06
Where and how to conduct OC/STH drug campaigns	41	5.3	Media	3	0.04
Other*	9	1.16			

**Notes**: *3 market, 1 each of phone, newspaper, urban people, husband, education, and family members.

The qualitative findings revealed that the participants received a variety of health information from the HEWs and volunteers.

Both Gare leaders and HEWs were transmitting the message to the community. There were no people who didn’t hear (P3, female, beneficiary, FGD, district).

HEWs and volunteers also stated that they provide a variety of health information to the community.

For instance, one participant stated

…we have been saying to the community taking the drugs alone is not useful, unless they keep their hygiene of personal, children, keep the sanitation of homes, toilet. …. (KII, HEW)

Moreover, another participant stated:“…But this new approach gave us an opportunity to provide health education during the campaign process as health extension workers fully engaged in the campaign” *(EGD, district, P2, WaSH Focal).*

### Median Score Comparison for SBCC Exposure

The average SBCC exposure score varied significantly across different groups, including study district, sex, age category, and marital status (p<0.05) (Table 3).

**Table 3:** Kruskal–Wallis Test and Mann–Whitney *U*-Test of SBCC Exposure Score with Socio-Demographic Characteristics

Parameters	Frequency	Kruskal–Wallis H	P-value
**Educational level**			
No formal education	523	2.9	0.229
Primary education	195		
Secondary education	58		
**Study district**			
O/Nada	173	45.8	<0.001
Gomma	157		
O/Beyam	149		
Kersa	147		
Manna	150		
**Age category (years)**			
15–24	102	11.9	0.008
25–34	234		
35–44	204		
≥45	236		
**Marital status**		11.3	0.003
Married	651		
Widowed	61		
Other	64		
**Mann–Whitney *U*-test**			
**Sex**		Z-value	
Female	552	−2.94	0.003
Male	224		

### Community Knowledge, Risk Perception, and Preventive Practices Toward OC

At baseline 83.4% of the respondents reported they had ever heard of OC, which increased to 97.3% after the intervention. The perceived causes of black flies at baseline were only 16.4%, which increased to 52.1% after the intervention. Finally, after the intervention there was an increase of 10.9% and 15.5% in the most common signs and symptoms of OC, respectively, which were intense skin itching and skin rash. The positive change in knowledge of blindness as a consequence of OC was as high as 51.2%, and its perceived severity was 15.3% from baseline. Taking an OC drug as a preventive measure increased by 34.3% from baseline (Table 4).The responses of “do not know” regarding the consequences and the mode of transmission decreases by 25% and 32% respectively at the end of the intervention (Supplementary Figures 2 and 3).

**Table 4:** Community Knowledge, Perception, and Practices Toward OC, Jimma Zone, 2021–22

Multidimensional Knowledge, Perception and Preventive Practices of OC	Baseline (N=732)		Endline (N=776)		% Change
	Frequency (n=yes)	(%)	Frequency (n=yes)	(%)	
Ever heard of OC	617	84.3	755	97.3	14
**Knowledge of OC**					
**High knowledge of signs and symptoms**	74	10.1	238	30.7	20.6
Intensive skin itching	508	623	80.3	10.9	10.6
Skin rash	334	474	61.1	15.5	16
Skin color change	77	268	34.5	24	24
Firm nodule in the skin	47	62	8.0	1.6	1.6
Eye itching	30	125	16.1	12	12
**High knowledge of mode of transmissions**	131	17.9	439	56.6	38.7
Blackfly bite	120	16.4	404	52.1	35.7
Swimming/washing with stream water	19	2.6	86	11.1	8.5
**High knowledge of consequences**	261	35.7	597	76.9	41.2
Social stigma	402	54.9	619	79.8	24.9
Skin scar/disfigure	343	46.9	480	61.9	15
Blindness	53	7.2	457	58.9	51.7
**High knowledge of preventive measures**	26	3.6	282	36.3	32.7
Taking an OC drug	320	43.7	605	78.0	34.3
Avoiding washing/contact with stream water	41	5.6	311	40.1	34.5
Use of chemical-treated bed net	9	1.2	112	14.4	13.2
Use of chemical spray	6	0.8	91	11.7	10.9
**High comprehensive knowledge of OC**	199	27.2	334	43.0	15.8
**Risk perception**					
**High risk perception**	370	50.5	449	57.9	7.4
OC is a severe disease	554	75.7	704	90.7	15
People who frequently touch running water are at risk of OC	327	44.7	464	59.8	15.1
A person living near running water is at high risk of getting OC	313	42.8	430	55.4	12.6
My families are at risk of OC infection	228	31.1	269	34.7	3.6
I am at risk of getting OC	227	31.0	266	34.3	3.3
**Preventive practice**					
**Practice at least one recommended preventive measure**	343	46.9	647	83.4	36.5
Taking OC drugs during the campaign	305	41.7	556	71.6	29.9
Avoiding washing with, swimming or touching river water	65	8.9	319	41.1	32.2
Sleeping under a chemical-treated bed net	15	2.0	112	14.4	12.4
Covering the body fully with clothes while touching river water	7	1.0	112	14.4	13.4
Spraying insecticide chemical	6	0.8	81	10.4	9.6

Generally, the comprehensive knowledge and risk perception of OC increased by 15.8% and 7.4%, respectively, from baseline. Regarding preventive measures and practices, the magnitude of do not know its prevention was decreased by 14.9 at the endline, whereas taking drugs during campaign and avoiding contact with river water increased by 29.9% and 32.2% at endline, respectively. The use of at least one preventive practice of OC increased by 36.5% from baseline (Table 5). However, many participants still used personal hygiene (55.9%) and environmental sanitation (46%) to prevent OC infection after the intervention (Supplementary Figures 4 and 5).

**Table 5:** One-Way ANOVA and Independent-Sample *t*-Test of Adjusted Key Outcome Variables Score Differences Towards OC with Socio-Demographic Characteristics

Parameters	Frequency (%)	Overall Knowledge		Risk Perception		Preventive Practice	
		F-Value	P-value	F-Value	P-value	F-Value	P-value
**Educational level**		1.809	0.165	1.072	0.343	5.312	0.005
No formal education	502(68.6)						
Primary education	177(24.2)						
Secondary education	53(7.2)						
**Study district**		9.13	<0.001	6.186	<0.001	24.277	<0.001
O/Nada	173(23.6)						
Gomma	114(15.6)						
O/Beyam	148(20.2)						
Kersa	147(20.1)						
Manna	150(20.5)						
**Age category (years)**		2.497	0.059	1.439	0.230	1.601	0.188
15–24	95(13)						
25–34	223(30.5)						
35–44	198(29)						
≥45	216(29.5)						
Independent-sample *t*-test							
**Sex**		t-value	P-value	t-value	P-value	t-value	P-value
Male	211(28.8)	1.28	0.199	1.49	0.136	2.5	0.012
Female	521(71.2)						

At the end of the assessment, the qualitative evidence revealed positive changes in community knowledge, perceptions, and preventive practices toward OC. The majority of FGD participants reported that it was transmitted by the bite of a black fly, had symptoms including body itching, and was prevented by taking the drug:

… the disease onko is caused by the black fly that comes from fast flowing water the major symptoms of the disease is body itching, leg swelling, etc. (P7, FGD, Youth, district)

Another participant mentioned:

Onchocerciasis a disease. It is transmitted to individuals when a black fly bites people. Even though I am not sure, I think the disease may catch any individual regardless of their age. We can prevent the disease by taking oncho drug (P3, FGD, female community member, district)

However, there were also misconceptions regarding the mode of transmission of OC. For instance, one FGD participant mentioned

….Separating night clothes and sleeping places, avoiding sharing of clothes, keeping personal hygiene”(P3, FGD, Youth, district).

Another FGD participant

said It [black fly] is found around non-flowing water bodies. It can also found in swampy area where wastes are disposed” (P6, Youth, district).

Furthermore, an additional participant stated

Onchocerciasis is a disease caused by a lack of personal and environmental hygiene” (P3, FGD, female youth, district).

Some FGD participants did not distinguish the disease from its medication or cause.For instance, one FGD participant mentioned that

Onchocerciasis is a small white medication (P6, female youth, district).

### Mean Overall Knowledge, Perception and Preventive Practices Score Comparison for OC

**Figure 1:**
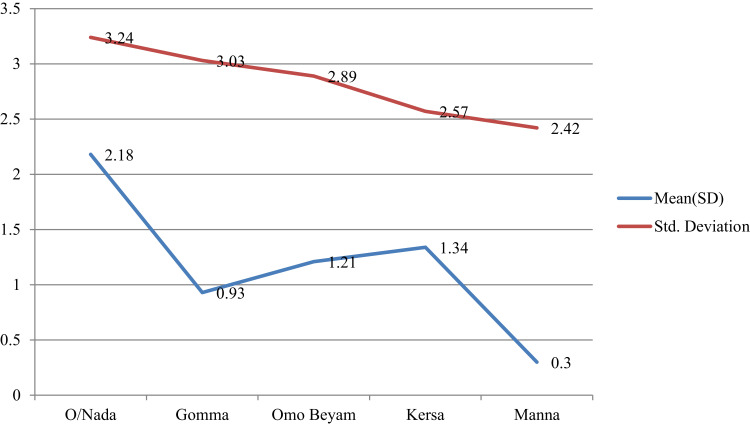
Mean plots of adjusted overall knowledge score difference at base and endline of OC per study districts in Jimma.

**Figure 2:**
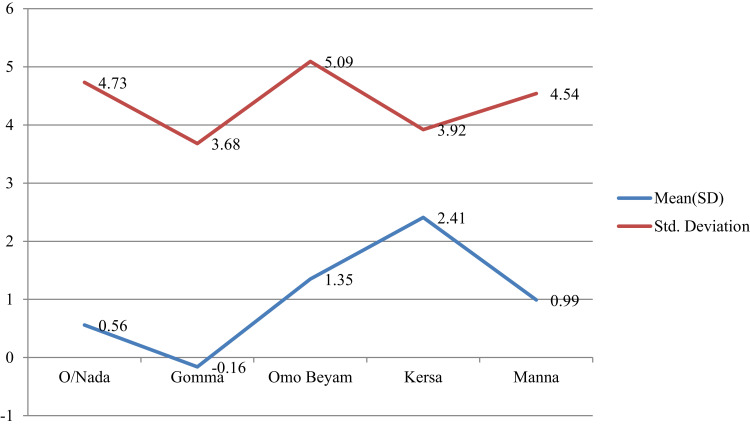
Mean plots of adjusted risk perception score difference at base and endline of OC per study districts in Jimma.

**Figure 3:**
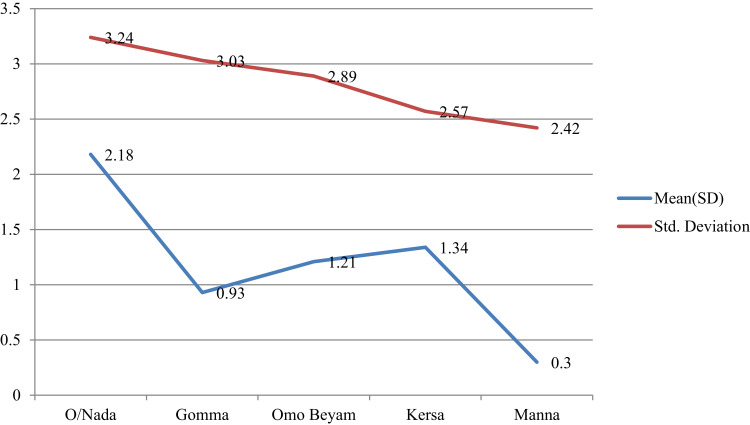
Mean plots of adjusted preventive practice score difference at base and endline of OC per study districts in Jimma.

The mean preventive practice scores varied among different groups based on educational level and between sexes and all were significantly among the groups for study district (p<0.05) (Table 5 and Figure 1–3).

**Figure 4:**
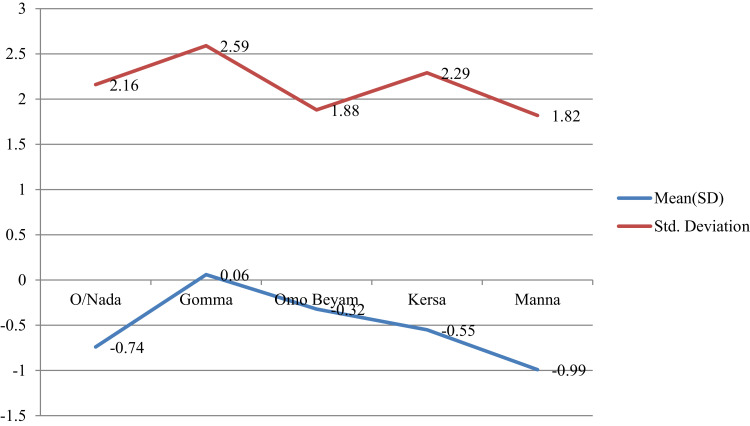
Mean plots of adjusted overall knowledge score difference at base and endline of STH per study districts in Jimma.

**Figure 5:**
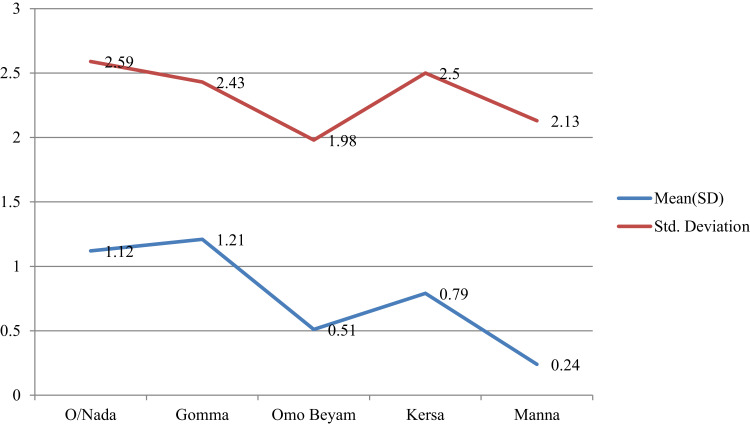
Mean plots of adjusted preventive practice score difference at base and endline of STH per study districts in Jimma.

### Knowledge, Preventive Practices, and Risk Perceptions About STH

Pre-school and school-age children were perceived to be most at risk for STH after the intervention, and the number of rural residents and pregnant women who perceived susceptibility to STH increased by 18.1% and 7.5%, respectively, at the endline (Supplementary Figure 6). The proportion of respondents who had heard of STHs slightly decreased from baseline by 2.3%, with its major perceived mode of transmission being through drinking contaminated water (73.4%) and eating contaminated food (71.4%), which decreased by half and nearly two-fifths (38.8%), respectively, after the intervention. Nearly half (50.7%) of the respondents perceived themselves as at risk of contracting STH, which decreased by 14% at the endline. The most commonly known symptoms of STH after the intervention were abdominal pain (86.9%) and loss of appetite (50%) (Table 6). At the end of the intervention, abdominal distension and irritability had increased by 15.7% and 8.5%, respectively (Supplementary Figure 7).

**Table 6:** Community Knowledge, Perception, and Practices of STH, Jimma, 2021–22

Multidimensional Knowledge, Perception, and Preventive Practices on STH	Baseline (N=732)		Endline (N=776)		Change (%)
	Frequency (n=yes)	(%)	Frequency (n=yes)	(%)	
Ever heard of STH	729	99.6	755	97.3	−2.3
**Knowledge of STH**					
**High knowledge of signs and symptoms**	362	49.5	337	43.4	−6.1
Abdominal pain	615	84.0	674	86.9	2.9
Nausea and vomiting	331	45.2	194	25.0	−20.2
Loss of appetite	317	43.3	388	50.0	6.7
Experience of diarrhea	296	40.4	359	46.3	5.9
Presence of parasite in the stool	197	26.9	113	14.6	−12.3
Feeling fatigue	36	4.9	116	14.9	10
Itching around anal area	19	2.6	76	9.8	7.2
**High knowledge of mode of transmission**	372	50.8	395	50.9	0.1
Drinking contaminated water	537	73.4	182	23.5	−49.9
Eating contaminated food	529	72.3	260	33.5	−38.8
Poor personal hygiene	306	41.8	418	53.9	12.1
Poor environmental sanitation	287	39.2	462	59.5	20.3
Eating raw or unwashed fruits and vegetables	126	17.2	106	13.7	−3.5
Preparing or eating food without washing hands	68	9.3	362	46.6	37.3
Lack of using a toilet	42	5.7	242	31.2	25.5
Walking barefoot	8	1.1	22	2.8	1.7
**High knowledge of protective measures**	348	47.5	444	57.2	9.7
Keeping food hygiene	468	63.9	235	30.3	−33.6
Keeping personal hygiene	371	50.7	352	45.4	−5.3
Taking medicine	284	38.8	139	17.9	−20.9
Washing hands before eating food	245	33.5	469	60.4	26.9
Washing hands before preparing food	225	30.7	524	67.5	36.8
Boiling drinking water	167	22.8	164	21.1	−1.7
Adding aquatab in drinking water	64	8.7	100	12.9	4.2
Proper utilization of a toilet	39	5.3	91	11.7	6.4
Wash hands after touching soil	26	3.6	156	20.1	16.5
Wearing shoes	5	0.7	60	7.7	7
**High knowledge of consequences**	366	50.0	333	42.9	−7.1
Stunting	319	43.6	577	74.4	30.8
Anemia	81	11.1	338	43.6	32.5
Poor school performance	7	1	50	6.4	5.4
Mental retardation	5	0.1	124	16.0	15.9
**High comprehensive knowledge of STH**	342	46.7	380	49	2.3
**Risk perception toward STH**					
**High risk perception**	404	55.2	312	40.2	−15
STH is a severe disease	636	86.9	734	94.6	7.7
My families are at risk of STHI	397	54.2	277	35.7	−18.5
Iam at risk of STHI	371	50.7	285	36.7	−14
**Preventive practices for STH**					
**High preventive practices**	325	44.4	389	50.1	5.7
Keeping food hygiene	550	75.1	600	77.3	2.2
Keeping personal hygiene	441	60.2	42.8	42.8	−17.4
Washing hands before eating/preparing food	234	32.0	503	64.8	32.8
Boiling drinking water	158	21.6	134	17.3	−4.3
Washing/cleaning fruit and vegetables before eating	139	19.0	94	12.1	−6.9
Adding aquatab to drinking water	57	7.8	150	19.3	11.5
Proper utilization of a latrine	38	5.2	116	14.9	9.7
Washing hands after soil contact	34	4.6	145	18.7	14.1

Three hundred and nineteen (43.6%) respondents mentioned stunting as a complication of STH, which increased by 30.8% at the endline. Generally, comprehensive knowledge increased by 2.6% from baseline and risk perception decreased by 15% from baseline. Respondents commonly practiced food hygiene (77.3%), personal hygiene (42.8%), and hand washing before eating or preparing food 64.8%, although 18.7% washed their hands after soil contact, and 14.9% properly utilized latrines after the intervention (Table 5). Knowledge of proper disposal of wastes as the STH preventive methods is increased by 10% after intervention. Moreover, practicing washing hands after toilet use and proper disposal of waste increased by 30.7% and 9.7%, respectively, at the endline (Supplementary Figures 8 and 9).

Qualitative finding revealed STH was mostly caused by drinking unclean water and contaminated food:

…in this area, it is known by name JARMII [germ]. … are mostly linked with drinking unclean water (P7, FGD, Youth, district)”.

Another FGD participant also stated:

Intestinal parasite is caused by drinking water that has no sanitation and hygiene. It can also be caused by eating contaminated food. Indeed, the intestinal parasite is also caused when individuals defecate on the bush. *(P3, Female youth, district)*

Most participants reported symptoms of STHI such as abdominal pain, nausea, and vomiting:

Once an individual gets intestinal parasites, the individual shows abdominal pain and abdominal bloating” (P4, Female youth FGD, district).

Another participant mentioned that“Nausea, vomiting, and abdominal bloating are also symptoms of intestinal parasites” *(P7, Female community member FGD, district).*

Most participants mentioned maintaining food and drinking water hygiene as preventive methods: “Keeping the hygiene of drinking water and eating food. Keeping environmental sanitation and hygiene” *(P1, Female youth FGD, district)*

Another participant also stated “proper utilization of latrine, personal and environmental sanitation” *(P3, Female community member FGD, district).*

### Mean Overall Knowledge and Preventive Score Comparison for STH

The average overall knowledge score and preventive practice towards STH showed significant variation among study district (p<0.05) (Table 7, Figures 4 and 5).

**Table 7:** One-Way ANOVA and Independent-Sample *t*-Test of Endline Overall Knowledge Score and Preventive Practice of STH with Socio-Demographic Characteristics

Parameters	Frequency (%)	Overall Knowledge		Preventive Practice	
		F-Value	P-value	F-Value	P-value
**Educational level**		1.781	0.169	0.521	0.594
No formal education	502(68.6)				
Primary education	177(24.2)				
Secondary education	53(7.2)				
**Study district**		4.591	0.001	4.331	0.002
O/Nada	173(23.6)				
Gomma	114(15.6)				
O/Beyam	148(20.2)				
Kersa	147(20.1)				
Manna	150(20.5)				
**Age category (years)**		0.042	0.989	1.301	0.273
15–24	95(13)				
25–34	223(30.5)				
35–44	198(29)				
≥45	216(29.5)				
**Role in the household**		0.618	0.539	0.802	0.449
house wife	515(70.4)				
Husband	171(23.4)				
Member	46(6.3)				
**Independent-sample *t*-test**					
**Sex**		t-value	P-value	t-value	P-value
Male	211(28.8)	−1.009	0.313	1.291	0.197
Female	521(71.2)				

### Median Risk Perception Score Comparison for STH

The median risk perception scores varied significantly among the groups of educational level, study district and marital status (p<0.05) (Table 8).

**Table 8:** Kruskal–Wallis Test and Mann–Whitney *U*-Test of Adjusted Risk Perception Score of STH with Socio-Demographic Characteristics

Parameters	Frequency (%)	Mean Rank	Kruskal–Wallis H	P-value
**Educational level**			9.8	0.007
No formal education	502(68.6)	377.87		
Primary education	177(24.2)	357.95		
Secondary education	53(7.2)	287.32		
**Study district**			22.013	<0.001
O/Nada	173(23.6)	363.16		
Gomma	114(15.6)	320.08		
O/Beyam	148(20.2)	402.75		
Kersa	147(20.1)	408.62		
Manna	150(20.5)	328.58		
**Age category (years)**			2.5	0.477
15–24	95(13)	340.08		
25–34	223(30.5)	373.03		
35–44	198(29)	377.22		
≥45	216(29.5)	361.56		
**Marital status**			7.684	0.021
Married	620(84.7)	375.38		
Widowed	54(7.4)	313.98		
Other	58(7.9)	320.49		
**Mann–Whitney *U*-test**				
**Sex**		Mean rank	Z-value	
Male	211(28.8)	370.56	−0.845	0.398
Female	521(71.2)	356.48		

### Changes in Mean Scores of Participant’s Pre and Post Intervention

Using Cohen’s d effect size (ES) measurements for OC, notable improvements in knowledge were observed, with effect sizes indicating medium to large effects, ranging from 0.54 to 1.14, excluding knowledge of symptoms. Risk perception showed a small change (ES = 0.32), while preventive practices improved moderately (ES = 0.54). For STH, knowledge of consequences demonstrated moderate improvement (ES = 0.69), whereas knowledge of prevention showed only a very small change (ES = 0.05). Other dimensions of knowledge regarding STH decreased overall. Additionally, preventive practices for STH increased (ES = 0.47), but risk perception declined from the baseline (Table 9).

**Table 9:** Changes in Mean Scores of Participants’ Pre and Post Intervention in Jimma, 2021–22

Key Outcome Variables	Mean (Standard Deviation)		Mean Difference	Pooled Standard Deviation	Cohen’s d
	Baseline (n=732	Endline (n=776)	Endline–Baseline		
**OC**					
Knowledge of symptoms	3.4(2.4)	4(2.0)	0.6	2.23	0.27
Knowledge of mode of transmission	0.95(2.0)	3.2(3.1)	2.25	2.46	0.92
Knowledge of consequences	3.63(2.8)	6.7(2.7)	3.07	2.75	1.11
Knowledge of prevention	1.28(1.5)	3.6(2.4)	2.32	2.19	1.06
Overall knowledge	3.5(2.2)	4.67(1.9)	1.17	2.03	0.58
Risk perception	4.51(3.35)	5.5(2.9)	0.99	3.08	0.32
Preventive practices	1.81(2.2)	3.04(2.4)	1.23	2.27	0.54
**STH**					
Knowledge of symptoms	4.94(2.1)	3.53(1.93)	−1.41	2.05	−0.69
Knowledge of mode of transmission	4.32(2.1)	3.31(1.92)	−1.01	2.02	−0.50
Knowledge of consequences	1.94(2.1)	3.51(2.38)	1.57	2.27	0.69
Knowledge of prevention methods	2.87(1.64)	2.95(1.67)	0.08	1.66	0.05
Overall knowledge	3.91(1.49)	3.36(1.53)	−0.55	1.51	−0.36
Risk perception	6.39(3.6)	5.56(3.2)	−0.83	3.40	−0.24
Preventive practices	2.56(1.51)	3.34(1.81)	0.78	1.65	0.47
Hand washing practice at critical times	24.28(3.85)	25.52(3.4)	1.24	3.62	0.34

### Wash Status

The proportion of participants with access to piped water for drinking increased from 14.8% (108) at baseline to 54.5% (423) at endline. Regarding water treatment practices, only 20.8% of the participants used water treatment at baseline, which increased to 49% after intervention. From the water treatment practices, boiling and using a water filter improved from 10% and 2.9% to 15.5% and 16.8% respectively (Table 10).

**Table 10:** Water Treatment Practices of Participants in Jimma, 2021–22

Characteristics	Baseline (n=732)		Endline (n=776)		% Change
Water Treatment Practices	Number (n=yes)	%	Number (n=yes)	%	
Using a water filter	18	2.9	153	19.7	16.8
Adding lemon to the drink water	16	2.6	150	19.3	16.7
Boiling and cooling	63	10.1	199	25.6	15.5
Filtering using clothes	28	4.5	117	15.1	10.6
Adding medicine like bishan gari, wuha agar, or aqua fresh	53	8.5	101	13.0	4.5
**Use at least one cleaning method**	152	20.8	380	49	28.2
**Other water cleaning practices**					
Covering the water storing equipment daily	223	35.7	227	29.3	−6.4
Daily cleaning of the water storing equipment	186	29.8	273	35.2	5.4
Do nothing	288	46.2	209	26.9	−19.3
Do not know	11	1.8	6	0.8	−1
Other*	6	0.08	1		

**Notes**: *baseline, 1 [adding salt, storing water, cleaning the drinking water environment, not storing water for a longer period, cleaning drinking instruments, letting it sit and filter]; end line, 1[clean water source].

The qualitative findings revealed a significant challenge in accessing adequate water for daily use. For instance, one FGD participant stated that “With water, it is difficult to get access to clean water in this rural area. May be boiling of drinking water can be a solution” *(FGD, P1, Youth, district).*

### Latrine Utilization and Its Perceived Benefits

Most of the participants (95.2%) had a toilet facility. The major perceived benefits of latrine reported were to protect family’s health and to prevent communicable diseases, which were increased after the intervention by 29.3% and 23.8% respectively (Table 11).

**Table 11:** Latrine Utilization and Its Perceived Benefit in Jimma, 2021_22

Characteristics	Baseline (n=732)		Endline (n=776)		Change (%)
Latrine status	(n=yes)	%	(n=yes)	%	
Private latrine	693	94.7	740	95.4	0.7
Shared latrine	7	1	39	5	4
Open defecation or on street	25	3.4	21	2.7	0.7
Neighbor’s toilet	18	2.5	10	1.3	1.2
**Perceived benefit of a latrine**					
Adequate knowledge	214	29.2	350	45.1	15.9
To protect families’ health	230	31.4	471	60.7	29.3
To prevent communicable disease	411	56.1	620	79.9	23.8
To prevent water contamination	21	2.9	70	9.0	6.1
To prevent different flies	352	48.1	307	39.6	−8.5
To keep privacy	103	14.1	113	14.6	0.5
To keep environmental and personal hygiene	464	63.4	368	47.4	−16
Do not know toilet benefits	9	1.2	5	0.6	−0.6

### Hand Washing Knowledge

The use of proper handwashing technique decreased by 3.4% at endline, and the major perceived benefits were to prevent COVID-19 and to prevent disease (Table 12).

**Table 12:** Hand Washing Knowledge of Participants in Jimma, 2021–22

Characteristics	Baseline (n=732)		Endline (n=776)		Change (%)
Perceived Benefit	Number (n=yes)	%	Number (n=yes)	%	
To prevent COVID-19	58	7.9	324	41.8	33.9
To prevent diseases	508	69.4	701	90.3	20.9
For personal comfort	43	5.9	72	9.3	3.4
To keep hygiene	608	83.1	539	69.5	−13.6
**Knowledge of proper hand washing**					
Washing with water and soap/ash	701	95.8	717	92.4	−3.4
Hand washing with water only	15	2.0	48	6.2	4.2
I do not know	10	1.4	6	0.8	−0.6
Washing five times a day	1	0.001	0	0	

### Handwashing Practice at Critical Times

At baseline, 88.5% of households always washed their hands after toilet use, increasing to 95.1% after the intervention. Additionally, hand washing after cleaning a child’s stool rose from 52.7% to 83.1% following the intervention and the frequency of hand washing after touching soil increased by 21.7% (Table 13). The overall adequate handwashing practice at baseline was 50.5% which increased to 64.9% after the intervention.

**Table 13:** Frequency of Hand Washing Practice at Critical Times in Jimma, 2021–22

Items	Always N (%)		Often N (%)		Sometimes N (%)		Never N (%)	
	Baseline	Endline	Baseline	Endline	Baseline	Endline	Baseline	Endline
After toilet use	648(88.5)	738(95.1)	56(7.7)	28(3.6)	23(3.1)	9(1.2)	5(0.7)	1(0.001)
After cleaning child’s stool	386(52.7	645(83.1)	56(7.7)	29(3.7)	15(2.0)	15(1.9)	275(37.6)	87(11.2)
Before preparing, touching, and serving meal	603(82.4)	616(79.4)	88(12.0)	148(19.1)	17(2.3)	8(1)	24(3.3)	4(0.5)
Before eating food or feeding child	593(81.0)	484(62.4)	87(11.9)	147(18.9)	22(3.0)	74(9.5)	30(4.1)	71(9.1)
After touching pets(dog, cat)	141(19.3)	233(30.0)	70(9.6)	89(11.5)	92(12.6)	180(23.2)	429(58.6)	274(35.3)
After cleaning dung	419(57.2)	537(69.2)	169(23.1)	107(13.8)	120(16.4)	71(9.1)	24(3.3)	61(7.9)
After touching soil	443(60.5)	345(44.5)	162(22.1)	121(15.6)	103(14.1)	278(35.8)	24(3.3)	32(4.1)
Before touching eyes, nose, and mouth	55(7.5)	125(16.1)	47(6.4)	75(9.7)	188(25.7)	307(39.6)	442(60.4)	269(34.7)

## Discussion

Although the MDA-based strategy targeting STH and OC has been in place for several years in Ethiopia to control and eventually eliminate these NTDs, challenges remain in achieving the desired MDA compliance. This is partly due to insufficient community awareness and preventive behaviors. To address this, it is crucial to develop well-designed, harmonized and culturally appropriate health education and communication activities that are integrated into the MDA efforts. Such initiatives should ensure local relevance and effectively engage communities in understanding and adopting preventive behaviors for the targeted NTDs. The current study examined the effectiveness of tailored SBCC interventions implemented before and during the MDA campaign in Jimma Zone. It focuses on enhancing community perception, knowledge, and preventive practices that align with local contexts through community engagement and appropriate communication channels. This study was intended to be the first of its kind in Ethiopia to examine the effectiveness of integrated SBCC interventions in MDA campaign on targeted NTDs among adults. Ethiopia has made plans for the elimination and control of both targeted NTDs by 2030, but the country’s progress toward this goal is sluggish, and both NTDs are widespread in the study area.^13^,^14^,^51^,^52^ The general population lacks adequate awareness regarding the diseases, and there is a significant gap in the design and implementation of behavior change strategies for NTD prevention compared to well-established drug treatment approaches.^53^

The study findings revealed that 88.8% of the communities were exposed to SBCC information. However, the reach of this information varied across different factors, including age, sex, and marital status. This SBCC intervention had a more significant impact on improving knowledge and preventive practices related to OC compared to STH. OC outcomes showed moderate to large effect sizes, indicating substantial improvements, while the impact on STH was more limited. The mean score for OC preventive practices varied by educational level and sex, and the median score for STH risk perception differed based on educational level and marital status. Furthermore, the scores for all outcomes varied among the study districts.

In this study, there was a significant increase in knowledge, perception, and preventive practices related to OC following the SBCC intervention. The highest effect size was for knowledge of consequences, and the knowledge of blindness as a result of OC increased significantly by 51.7%, which seemed to result in an increase of 15% in the perceived severity of OC. These findings highlight the crucial role of SBCC in dispelling misconceptions about OC and enhancing health literacy, representing a positive advance toward the control and elimination of the disease. This aligns with previous research conducted in Enugu State, Southern Nigeria, which also reported substantial improvements in OC knowledge, attitudes, and practices following a health education program.^54^ Furthermore, earlier studies have shown that group communication strategies, a key component of SBCC, are more effective in improving knowledge and perceptions among farmworkers compared to individual approaches.^55^

The current study found an even more pronounced increase in the proportion of respondents correctly attributing the cause of OC to blackflies, from 16.1% at baseline to 52.1% post-intervention. This level of improvement in understanding the mode of OC transmission was higher than that observed in the Enugu State study.^54^ A possible explanation for this stronger impact could be differences in the sample size, study duration, or the specific components included within the SBCC intervention package between the two studies. However, the current study also found that certain misconceptions about OC transmission persisted even after the intervention. Specifically, nearly half (45.4%) of respondents continued to believe that OC is caused by the exchange of clothes, and one-third (33.8%) still attributed it to poor personal hygiene. This finding aligns with the observations from the Enugu State study, which similarly reported the persistence of some misconceptions related to OC causation even after the health education program.^54^

The key findings regarding knowledge of preventive measures are positive. The study shows that awareness of the recommended preventive measures, such as taking the OC drug (Ivermectin) and avoiding contact with fast-flowing rivers, improved significantly by around 34% from the baseline. In the current study more than three-quarters (78%) of the respondents knew that Ivermectin was the drug for the treatment of OC. This finding is in line with an earlier study conducted in Enugu, Nigeria, which reported that Ivermectin was the major preventive measure known within the communities.^54^ Knowledge plays a crucial role in changing health-related behaviors; a solid understanding of the disease promotes preventive practices among at-risk populations and enhances adherence to control programs.^56^

Taking the OC drug as a preventive practice increased significantly, by 29.9%, which is a promising outcome. The proportion of taking medication as a preventive practice was 71.6% at the endline, which was still less than the national goal. To effectively control and eliminate OC, it is essential to achieve complete geographic coverage and maintain consistently high coverage of Ivermectin treatment. A minimum coverage rate of 80% for at least 12 to 15 years is necessary to ensure the treatment reaches all at-risk populations within the affected areas, reducing the transmission of the disease.^57^ Theoretically, the extended parallel process model suggests that one can anticipate corresponding behavioral uptake if messages are received positively, in support of danger control.^58^ Furthermore, this result is consistent with the theoretical foundation of performance and knowledge, which supports the idea that behavioural change interventions aimed at improving threat perceptions through well-balanced coping mechanisms would be successful.^59^

It is promising to note that, in addition to MDA, complementary control and prevention strategies for OC in endemic areas have shown significant improvements following intervention. For example, the practice of avoiding contact with stream water increased by 32% at the endline. One potential contributing factor to this positive outcome could be the effective implementation of SBCC interventions. By involving key stakeholders, including opinion leaders, the SBCC approach may have improved the community’s acceptance and adoption of the recommended preventive actions. One of the primary preventive efforts for OC is public education on the disease and its preventive measures to promote behavior and social change toward the prevention of its infection. As knowledge is regarded as a fundamental component of the process of health behavior change efforts, the findings suggest that an intervention aimed at promoting comprehensive knowledge would result in effective behavior change outcomes in OC. Thus, we recommend health education and community engagement initiatives should be started early in the targeted NTD MDA campaign.

Statistical analysis revealed a significant variation in the mean score differences of preventive practices for OC across different educational levels. This variation may stem from differences in health literacy and access to information, indicating that individuals with varying educational backgrounds possess differing levels of knowledge and implementation of preventive practices. Furthermore, the mean score differences at both baseline and endline varied between sexes. This disparity could be attributed to differences in social and cultural norms, as well as varying access to health information and education for males and females.

The current study revealed that almost all respondents had heard of STH with slight decrement compared to baseline. However, when they were further asked about SBCC exposure, most of the participants reported receiving education on the benefits of the OC drug and co-administration of the two drugs for STH and OC. Regarding STH and OC, less than 20% (18% and 14.6% for OC and STH respectively) reported hearing about them during the intervention.

Regarding STH related knowledge change revealed that the intervention had a moderate effect on increasing knowledge about the consequences of STH infections. This suggests that participants gained a significant understanding of the potential outcomes or effects of STH infections after the intervention. In contrast, the intervention had a very small effect size (0.05) on increasing knowledge about prevention measures for STH infections. However, when expressed as a percentage, this translates to a 9.7% increment in knowledge, indicating that while the effect size is small, there was still a notable increase in knowledge about how to prevent STH infections.

In fact, there was also a significant increase in the proportion of respondents who correctly identified preparing or eating food without washing hands and the lack of a toilet as modes of transmission for STH. Specifically, after the intervention, the percentage of respondents mentioning these behaviors as modes of transmission increased by 37.3% and 25.5% respectively. This finding suggests that the intervention was successful in improving the knowledge and awareness of respondents regarding the transmission routes of STH, particularly in relation to the importance of hand hygiene and access to proper sanitation facilities. However, the comprehensive knowledge of STH was not increased. For the purpose of directing control strategies to initiate sufficient changes in perceptions and preventive practices toward its control and ultimate eradication, we suggest that the knowledge measure should be comprehensive.

The risk perception of STH decreased significantly from baseline, which may have been caused by improved protective measures, dispelling of misconceptions, and increased knowledge. Perceptions and general public awareness may impact the community’s decision-making regarding drug intake and the creation of interventions for the implementation of MDA for eligible community members. On the other hand, preventive practices for STH increased after the intervention (ES=0.47), which showed that the intervention had a promising impact. For example, there was a significant increase in the proportion of respondents mentioning washing their hands after toilet use and washing their hands before eating or preparing food, which increased by 30.7% and 32.8%, respectively, at the endline. Notably, at baseline, 8.1% of the participants reported taking no preventive measures against STH. However, following the intervention, this percentage significantly decreased to 0.4%, indicating a marked improvement in participants’ adoption of preventive practices. Our findings highlight the importance of health education as a cost-effective strategy for ensuring a sustainable and effective STH control program.^60^ Our finding is consistent with existing research, which has demonstrated that educational interventions can lead to significant improvements in knowledge and practice related to STH. For instance, studies in Malaysia have shown that educational interventions resulted in substantial enhancements in both knowledge and practice among participants.^61^ Similarly, a study in Bangladesh found that such an intervention effectively improved community awareness about STH.^62^

Generally, the findings regarding STH related changes following the intervention were generally not as promising as the results for OC. This may be due to a few factors: differences in the exposure to SBCC activities between the two diseases, the longer duration of control measures for OC in the study area compared to STH, and perceptions of susceptibility to STH infections. The qualitative insights and survey results indicate that many people believe STH is a disease that only affects children, and at the end of the survey, less than 1% of respondents believed that adults were more susceptible to STH. However, there were some improvements observed, with the perceptions of susceptibility to STH increasing by 7.5% among pregnant women and 18.1% among rural adults by the end of the study, suggesting some positive impact. Another possible explanation for the lower change in STH-related outcomes compared to OC is that the intervention was delivered for both diseases simultaneously, and as the consequences of OC, such as blindness, are more severe, people may have paid more attention to OC-related messages and activities. Additionally, perceptions of eligibility for MDA may have also influenced the attention given to the intervention for each disease. Future studies should consider separating the interventions for each disease to confirm these findings.

Statistical analysis on comparison of score variation by Sociodemographic characteristics revealed risk perception of STH was varied by educational level and marital status. The possible reasons could be individuals with higher educational attainment and married individuals tended to have a higher risk perception of STH infections, likely due to better access to health information, healthcare services, and a greater sense of responsibility towards others. This suggests that individuals with different educational backgrounds and marital statuses have distinct levels of awareness and understanding of the risks associated with STH infections. Finally, the mean score differences in overall knowledge, perceptions, and preventive practices for both STH and OC was varied among the study districts. Possible reasons for these district-wise variations may include differences in exposure to SBCC, the disease’s endemicity, the extent of control measures implemented, the severity of the disease, participants’ educational levels, and access to information. In general, the results of this study suggest that considering these factors is important when designing and implementing health education programs.

Evidence indicates that altering one’s behavior is “critical to developing sustainable services and optimizing the public health benefits of investment in water and sanitation”.^63^ “Toilets might not be used, water could still be contaminated, food will continue to be polluted, and dignity will be compromised”, as stated by Water Aid, illustrating that merely expanding access to WASH infrastructure does not ensure a decline in the prevalence of disease in the absence of changes in WASH behavior.^64^ In this study, hand washing behavior at critical times, perceived benefit of toilet use, and water treatment practices significantly increased after the intervention, which was corroborated by earlier studies.^65^,^66^ Water treatment practice, knowledge of the perceived benefit of toilets, and hand-washing practice increased by 28.2%, 15.9% and 14.4%, respectively. This shows how vital health education is in shaping community understanding, creating appropriate perceptions, encouraging the adoption of recommended preventive measures, and serving as a successful NTD control strategies. Indeed, based on the findings of the study, it is evident that there is a need for the campaign delivery team and relevant organizations to strengthen the education program in preparation for and during the MDA campaign targeting NTDs. Strengthening the education program is crucial for ensuring the successful control and eventual elimination of these diseases.

## Strengths and Limitations of the Study

This study has several strengths that contribute to its robustness. These strengths include a large sample size, which enhances statistical power and increases the generalizability of the findings. The use of a mixed design allows for a comprehensive understanding of the issue by combining quantitative and qualitative data. Additionally, the intervention itself was tailored and utilized various communication channels, increasing the effectiveness of the intervention in reaching and engaging the target population.

The limitations of this study include the absence of a control group; therefore, baseline data were used as a control for the endline. The use of self-reported data, which may have introduced an information bias, is another limitation. However, the data were collected anonymously, which lessens the possibility of this kind of bias. Finally, because the intervention period was short, we were unable to ensure that its effects would continue in the long term. These limitations highlight areas for improvement in future research, including the inclusion of control groups, more rigorous data collection methods, and longer intervention periods to assess sustainability of effects over time.

## Conclusion

The study found that 88.8% of the communities were exposed to the SBCC information. Importantly, the SBCC intervention had a greater impact on improving knowledge and preventive practices related to OC, with moderate to large effect sizes, compared to a more limited impact on STH. The mean score for OC preventive practices varied by educational level and sex, while the median score for STH risk perception differed based on educational level and marital status. Furthermore, the scores for all outcomes were observed to vary across the study districts. Additionally, the study found improvements in handwashing practices at critical times following the SBCC intervention. These findings highlight the need to consider these factors while designing educational interventions.

The findings highlight that the integration of SBCC into MDA is promising with respect to improving the communities’ knowledge, perception, and preventive practices on OC and preventive practices and knowledge of consequences toward STH. The results emphasize the need for campaign delivery teams and relevant stakeholders to prioritize and strengthen education programs, both during and prior to MDA campaigns. By adopting a comprehensive approach, SBCC strategies can be tailored to the specific needs and characteristics of diverse communities, ultimately maximizing the impact and effectiveness of integrated SBCC interventions in MDA.

## Data Availability

The data used in this analysis can be obtained from the corresponding author upon request.

## Abbreviations

ES: Effect size; MDA: Mass drug administration; NTD: Neglected tropical diseases; OC: Onchocerciasis; SBCC: Social and behavioral change communication; STH: Soil transmitted helminthes; WaSH: Water, sanitation and hygiene.
